# Metformin does not decrease the incidence of shoulder arthroplasty in patients with glenohumeral joint osteoarthritis

**DOI:** 10.1016/j.xrrt.2025.07.006

**Published:** 2025-08-05

**Authors:** Tarek Haj Shehadeh, Jawaad Chaudhry, Ahmed Abdeen, Gary Updegrove, April Armstrong, Nathan Lanham

**Affiliations:** Department of Orthopedics and Rehabilitation, Penn State Health, Penn State Milton S. Hershey Medical Center, Hershey, PA, USA

**Keywords:** Shoulder arthroplasty, Metformin, Antidiabetics, Prognosis, Diabetes mellitus, Osteoarthritis, Glenohumeral joint

## Abstract

**Background:**

Metformin is an antidiabetic medication that is routinely prescribed. Recent reports suggest a dose-response disease-modifying effect on osteoarthritis (OA) of the knee with patients on metformin having a lower incidence of total knee arthroplasty. However, it is unknown whether this effect translates to OA of the shoulder. Therefore, the purpose of this study is to investigate the effect of metformin on the likelihood of having shoulder arthroplasty in patients with shoulder OA.

**Methods:**

The TriNetX database was used to identify a cohort of patients with shoulder OA. Two groups were formed. The first group was comprised of patients who were not prescribed metformin. The second group was comprised of patients who were prescribed metformin. Propensity score matching was performed to control for multiple cofounding variables including hemoglobin A1C levels. Both matched cohorts consisted of 26,327 patients each. The outcomes of interest were the incidence of shoulder arthroplasty up to 10 years after the diagnosis of shoulder OA, as well as serum inflammatory marker levels (C-reactive protein (CRP), erythrocyte sedimentation rate (ESR), and Cortisol).

**Results:**

A total of 1,245 (4.729% risk) patients in the metformin group underwent shoulder arthroplasty compared to 962 (3.654% risk) patients in the nonmetformin group. The metformin group had averages of 30.703 mg/L ± 57.155 mg/L for CRP, 33.692 mm/hour ± 27.857 mm/hour for ESR and 13.224 μg/dL ± 8.450 μg/dL for cortisol. The nonmetformin group had averages of 36.577 mg/L ± 60.627 mg/L for CRP, 5.574 mm/hour ± 29.719 mm/hour for ESR and 13.326 μg/dL ± 8.571 μg/dL for cortisol. The metformin group had a significantly increased risk of arthroplasty of 1.075% [95% confidence interval (0.733%, 1.417%), *P* < .0001 and odds ratio = 1.309 [95% confidence interval (1.201-1.426)] compared to the nonmetformin group and had a longer follow-up period (*P* < .0001). Mean ESR and CRP inflammatory markers levels in the metformin group were significantly lower than the nonmetformin group (both *P* < .0001) However, there was a nonsignificant decrease in average cortisol levels (*P* = .779) in the metformin group when compared to the nonmetformin group.

**Discussion/Conclusion:**

Patients with shoulder OA who are prescribed metformin are more likely to undergo shoulder arthroplasty than those not taking metformin, despite having significant decreases in inflammatory marker levels. These findings stand in contrast to previous data which suggest a potential dose-response disease-modifying effect on OA of the knee. Additional research is needed to better elucidate the mechanisms of metformin on OA.

Shoulder osteoarthritis (OA) is a debilitating disease that causes shoulder pain, restricted joint motion, and affects up to 20% of adults over the age of 60.^1^^,^^21^ Shoulder OA is treated in a variety of ways, including shoulder arthroplasty for end-stage disease progression.^6^ Shoulder arthroplasty is one of the fastest-growing procedures and is expected to increase exponentially over the coming decades.^6^ Despite the prevalence of shoulder OA and the expected increase in shoulder arthroplasty, there is a relative paucity of literature on shoulder OA and related diseases as compared to hip and knee OA.

Metformin is a first-line drug therapy for diabetes that lowers glucose levels and improves metabolic health. Recent studies suggest that metformin possesses anti-inflammatory properties that may play a role in limiting OA-related pain and disease progression in the knee.^8^^,^^14^, ^15^, ^16^ Inflammation is a key pathophysiological process in the development and progression of OA.^14^ The process of inflammation in OA occurs through a myriad of cascades and pathways. The adenosine monophosphate-activated protein kinase (AMPK) pathway has been shown to be critical in maintaining the joint homeostasis of chondrocytes. Downregulation of AMPK activity was linked to further progression of OA.^4^ Metformin was found to inhibit complex I of the mitochondrial respiratory chain and increase AMPK activity. Through this pathway, metformin is believed to decrease inflammation, limit chondrocyte apoptosis, and ameliorate pain associated with OA progression.^19^

These purported disease-modifying effects of metformin on knee OA have not been examined with respect to shoulder OA. Therefore, the purpose of this study is to explore the association between metformin use in shoulder OA and the likelihood of undergoing shoulder arthroplasty. We hypothesize that patients on metformin will have a lower incidence of shoulder arthroplasty when compared to controls who have shoulder OA and are not on metformin.

## Methods

This study used the TriNetX database, which harbors deidentified data of more than 100 million patients from over 90 health-care organizations. As a result, this study was exempted from institutional review board approval. Patient populations, characteristics, comorbidities, and outcomes of interest were identified using International Classification of Diseases 10, Current Procedural Terminology, and Systematized Nomenclature of Medicine codes.

We queried the database using the Research Network for patients older than 18 years of age who were diagnosed with shoulder OA between January 2014 and January 2024 and formed 2 cohorts: patients with shoulder OA on metformin (32,225 patients) and patients with shoulder OA who were not taking metformin (861,013 patients—control group). We excluded patients with proximal humeral fractures. Subsequently, a 1:1 propensity score matching was performed using the TriNetX built-in function to control for multiple patient- and disease-related factors. This resulted in 26,327 patients in each cohort. Table I shows the demographics of the 2 cohorts after propensity matching. Adopting a cutoff of standardized difference of at 0.1 to assess balance,^12^ our cohorts are well-balanced across all included factors after propensity score matching. The outcomes of interest were shoulder arthroplasty and levels of inflammatory markers (C-reactive protein (CRP)/erythrocyte sedimentation rate (ESR)/cortisol) up to 10 years after shoulder OA diagnosis.

**Table I:** Characteristics of both cohorts after 1:1 propensity score matching.

Cohort	Characteristic	Mean ± SD	Patients	% Of cohort	Std diff.
Demographics					
Metf +	Age at Index	65.1 ± 10.5	26,327	100%	0.014
Metf −		65.0 ± 11.3	26,327	100%	
Metf +	Female		12,518	47.50%	0.005
Metf −			12,448	47.30%	
Metf +	Male		12,643	48.00%	0.019
Metf −			12,899	49.00%	
Diagnosis					
Metf +	Disorders of lipoprotein metabolism and other lipidemias		20,537	78.00%	0.048
Metf −			20,007	76.00%	
Metf +	Overweight, obesity and other hyperalimentation		11,884	45.10%	0.021
Metf −			11,604	44.10%	
Metf +	Hypertensive diseases		21,692	82.40%	0.045
Metf −			21,229	80.60%	
Metf +	Ischemic heart diseases		8,286	31.50%	0.034
Metf −			7,879	29.90%	
Metf +	Type 2 diabetes mellitus		23,754	90.20%	0.002
Metf −			23,768	90.30%	
Metf +	Diseases of liver		4,345	16.50%	0.035
Metf −			4,004	15.20%	
Metf +	Heart failure		4,343	16.50%	0.036
Metf −			4,000	15.20%	
Metf +	Chronic kidney disease		5,752	21.80%	0.004
Metf −			5,706	21.70%	

Cohort	Hemoglobin A1c % level	Patients	% of Cohort	Std diff.
Laboratory				
Metf +	<7.50%	16,579	63.00%	0.024
Metf −		16,278	61.80%	
Metf +	>7.50%	14,421	54.80%	0.006
Metf −		14,346	54.50%	

*Metf+*, Metformin group; *Metf−*, nonmetformin group; *SD*, standard deviation.

For statistical analysis, we used the built-in analysis setup in the TriNetX system with a significance cutoff value set at α < 0.05.

All codes used for the database query are included in Supplementary Table S1.

## Results

The mean follow-up duration was 3.9 ± 2.5 years for the metformin group compared to 3.7 years ± 2.5 years for the nonmetformin group. A total of 1,245 (4.729% risk) patients in the metformin group underwent shoulder arthroplasty compared to 962 (3.654% risk) patients in the nonmetformin group. The metformin group had averages of 30.7 mg/L ± 57.2 mg/L for CRP (reference range: <10 mg/L), 33.7 mm/hour ± 27.9 mm/hour for ESR (reference range varies by age) and 13.2 μg/dL ± 8.5 μg/dL for cortisol (reference range: 4.1-4.7 μg/dL). The nonmetformin group had averages of 36.6 mg/L ± 60.6 mg/L for CRP, 35.6 mm/h ± 29.7 mm/h for ESR, and 13.3 μg/dL ± 8.5 μg/dL for cortisol.

The metformin group had a significantly increased risk of arthroplasty of 1.075% [95% confidence interval (0.733%, 1.417%), *P* < .0001 and an odds ratio = 1.309 [95% confidence interval (1.201-1.426)] compared to the nonmetformin group and had a longer follow-up period (*P* < .0001). Mean CRP and ESR inflammatory markers levels in the metformin group were significantly lower than the nonmetformin group (t = −5.582, *P* < .0001; t = −3.835, *P* < .0001, respectively) (Table II). However, there was a nonsignificant decrease in average cortisol levels (t = −0.281, *P* = .779) in the metformin group when compared to the nonmetformin group.

**Table II:** Difference in mean inflammatory marker levels between the 2 cohorts.

Group	Metformin	Nonmetformin	t	*P* value
CRP (% of cohort)	30.7 ± 57.2 (24.39%)	36.6 ± 60.6 (23.21%)	−5.582	<.0001
ESR (% of cohort)	33.7 ± 27.9 (26.74%)	35.6 ± 29.7 (25.21%)	−3.835	<.0001
Cortisol (% of cohort)	13.2 ± 8.5 (4.14%)	13.3 ± 8.6 (4.21%)	−0.281	.779

*CRP*, C-reactive protein; *ESR*, Erythrocyte sedimentation rate.

Significance *P* < .05.

## Discussion

The most notable finding of the present study was that metformin use was associated with an increased incidence of shoulder arthroplasty. Conversely, patients not on metformin were less likely to undergo a shoulder arthroplasty. These results conflict with existing literature,^5^^,^^16^^,^^17^^,^^19^ which has suggested that the anti-inflammatory and disease-modifying effects of metformin may decrease the incidence of total knee and total hip arthroplasty in patients with hip and knee OA.^8^^,^^15^ Interestingly, metformin use in the present study was associated with decreased ESR and CRP inflammatory markers. However, this decrease in inflammatory markers was not associated with a decreased incidence of shoulder arthroplasty.

Multiple studies explore the effects of metformin on OA. Xu et al found that metformin decreased OA progression in a rat OA model by modulating the PI3K/AKT/mTOR signaling pathway.^22^ Similarly, Zou et al showed that metformin improved chondrocyte ferroptosis sensitivity via the AMPK/ACC signaling pathway and diminished overall joint inflammation in the OA mouse model.^24^ In human patients, metformin use has been associated with a decreased incidence of total hip arthroplasty.^15^ Furthermore, metformin use has been associated with significantly improved pain, decreased stiffness, and improved function when compared to placebo in hip and knee OA populations.^3^^,^^4^

In addition, several studies discussed the immune modulatory effects of metformin, which may be beneficial in slowing the progression of joint degeneration. Son et al observed within *in vitro* murine models that metformin can inhibit Th17 cell differentiation while increasing Treg differentiation. Additionally, they observed *in vivo* collagen-induced arthritis mice treated with metformin had a significantly decreased ability to perform osteoclastogenesis.^18^ In the context of clinical application, Aiad et al found that obese patients with knee OA on metformin had a significant decrease in their Western Ontario and McMaster Universities Arthritis Index total scale. These results indicate that metformin may have a role in reducing the rate and severity of joint degeneration.^2^

The effects of anti-inflammatory medications on the rates of joint arthroplasty are unclear. There is some evidence indicating that patients on non-steroidal anti-inflammatory drugs are at a higher risk of undergoing total knee arthroplasty.^9^ Although this may be associated with the alleviation of symptoms thus allowing the joint degeneration to progress unnoticed by the patient and not directly associated with the anti-inflammatory properties of the therapy. In addition, there is conflicting evidence on whether non-steroidal anti-inflammatory drugs affect the progression of OA.^11^

Along the same line, the association between inflammatory markers and the incidence of joint arthroplasty is also unclear. For instance, high-sensitivity CRP was significantly associated with increased pain scores in patients with hip or knee OA.^20^ Another systematic review showed that high-sensitivity CRP levels were not associated with OA progression, but had a weak but significant correlation with pain scores.^13^ Another systematic review found a similar finding of a positive association between CRP levels and pain.^10^ As such, it could be hypothesized that decreased inflammatory markers could lead to decreased pain and subsequently decreased need for joint replacement.

There are several possible explanations for the present study's results. First, metformin's influence on systemic inflammation may not have prevented the progression of shoulder OA nor the resulting symptoms caused by shoulder OA. Although metformin was associated with a decrease in ESR and CRP, it did not significantly decrease cortisol levels. Thus, it may be that a more significant decrease in these inflammatory markers would have been associated with a decreased incidence of shoulder arthroplasty. Furthermore, other indications for shoulder arthroplasty, such as cuff tear arthropathy and massive cuff tears, are less likely to be affected by inflammation/inflammatory marker levels compared to primary shoulder OA. In addition, the associated symptomatic improvement with metformin could lead to greater ease in using the affected joint, which may in turn increase degenerative pathology and thus increase the incidence of total shoulder arthroplasty. Second, it is possible that the cohort that was on metformin had more severe shoulder OA which resulted in an increased incidence of shoulder arthroplasty. We were not able to control for the severity of shoulder OA. Third, dosing and metformin therapy compliance data were not available for analysis. Therefore, it is possible that patients were on different doses of metformin and/or not taking metformin as prescribed, since metformin has been shown to have dose-related disease-modifying effects.^7^ Fourth, it is possible that the codes used did not distinguish between acromioclavicular joint OA and glenohumeral joint OA. Finally, it is possible that the severity of diabetes influenced the incidence of shoulder arthroplasty, since diabetes is a known risk factor for increased postoperative complications, such as delayed wound healing and infections.^23^ Therefore, patients with more severe uncontrolled diabetes were not deemed medically optimized for surgery and thus did not have the same incidence of shoulder arthroplasty. Although we attempted to control for the severity of diabetes by using threshold hemoglobin A1c data, the metformin group likely had patients with more severe uncontrolled diabetes.

There are multiple limitations to the present study. First, the indication for shoulder arthroplasty was not specifically designated in within the data. Therefore, arthroplasty indications for diagnoses other than shoulder OA were not directly captured and may have influenced the incidence of shoulder arthroplasty. In addition, inflammatory markers were not available for all the patients, and autoimmune diseases associated with increased inflammatory markers such as rheumatoid arthritis and systemic lupus erythematosus were not accounted for in the propensity matching, nor systemic glucocorticosteroid intake. Finally, the metformin group was followed up for a longer duration, which could explain the increased incidence in the metformin group.

## Conclusion

Metformin was associated with an increased incidence of shoulder arthroplasty despite being associated with decreased levels of inflammatory markers. Further research is needed to better evaluate metformin's disease-modifying effects and the incidence of shoulder arthroplasty, as well as the association between inflammatory marker levels and OA progression.

## Disclaimers

Funding: No funding was disclosed by the authors.

Conflicts of interest: Dr Updegrove received Consultant fees and royalties from Aevumed, is a paid consultant to Stryker and DePuy Synthes, and is a paid consultant to and received royalties from Globus. Dr Armstrong received Consultant fees and royalties from Aevumed and Consultant fees and royalties from Zimmer Biomet. The other authors, their immediate families, and any research foundations with which they are affiliated have not received any financial payments or other benefits from any commercial entity related to the subject of this article.
