# Supplementary material for: Metformin does not decrease the incidence of shoulder arthroplasty in patients with glenohumeral joint osteoarthritis
Source: JSES Rev Rep Tech. 2025 Aug 5;5(4):621–4. doi: 10.1016/j.xrrt.2025.07.006 (PMC12573443; doi:10.1016/j.xrrt.2025.07.006)
Supplement: Supplementary Table S1 [file mmc1.docx]

Supplementary Table 1: Table presenting the diagnostic codes used to query the database.

*ICD-10: International Classification of Diseases; CPT: Current Procedural Terminology; TNX: TriNetX Proprietary Code System*

| **Diagnoses** | **Code** |
| --- | --- |
| Primary osteoarthritis, shoulder | ICD10 - M19.01 |
| Post-traumatic osteoarthritis, shoulder | ICD10 - M19.11 |
| Secondary osteoarthritis, shoulder | ICD10 - M19.21 |
| Fracture of upper end of humerus | ICD10 - S42.2 |
| Pathological fracture, humerus | ICD10 - M84.42 |
| metformin | RXNORM - 6809 |
|  |  |
| **Outcomes** | **Code** |
| Shoulder arthroplasty | CPT - 1004224; ICD10 - 0RRJ00Z, 0RRJ07Z, 0RRJ0JZ, 0RRJ0KZ, 0RRK00Z, 0RRK07Z, 0RRK0JZ, 0RRK0KZ; SNOMED - 42262007, 733429004 |
| C-reactive Protein | TNX - 9063 |
| Erythrocyte sedimentation rate | TNX - 9066 |
| Cortisol | TNX - 9035 |
